# Early mobilisation after total hip or knee arthroplasty: A multicentre prospective observational study

**DOI:** 10.1371/journal.pone.0179820

**Published:** 2017-06-27

**Authors:** Matthew J. Chua, Andrew J. Hart, Rajat Mittal, Ian A. Harris, Wei Xuan, Justine M. Naylor

**Affiliations:** 1 University of New South Wales, Sydney, New South Wales, Australia; 2 Whitlam Orthopaedic Research Centre, Liverpool Hospital, Sydney, New South Wales, Australia; 3 Ingham Institute of Applied Medical Research, Sydney, New South Wales, Australia; Georgia Regents University, UNITED STATES

## Abstract

**Objective:**

Early mobilisation is recommended following total hip arthroplasty (THA) or total knee arthroplasty (TKA) to prevent venous thromboembolism (VTE). We sought to determine the proportions of patients that first mobilised on post-operative day 0 (POD 0) and factors associated with earlier time to mobilisation.

**Methods:**

A prospective cohort study was conducted involving patients with hip or knee osteoarthritis who had undergone primary unilateral THA (n = 818) and TKA (n = 989) at 19 Australian hospitals. Patient-related (e.g. age, gender, body mass index), treatment-related (e.g. hospital site, presence of indwelling catheter) and mobilisation-related variables were collected on standardised forms. Time was measured by post-operative days, where POD 0 was defined as the day of surgery ending at midnight. Multivariate Poisson regression analysis identified associations between patient- and treatment-related covariates and time to mobilisation.

**Results:**

Inter-hospital variation was evident, but overall, only 9.4% of THA and 5.6% of TKA patients mobilised on POD 0. For THA patients, earlier time to mobilisation was associated with hospital site and absences of an indwelling catheter and acute complications. For TKA patients, earlier time to mobilisation was associated with hospital site and absence of donor blood transfusion.

**Conclusions:**

Few THA and TKA patients mobilise POD 0, although some hospitals appear more aggressive with their mobilisation attempts than others. Treatment-related factors, not patient-related, are associated with post-operative day of mobilisation, indicating the potentially pivotal role of service providers in promoting early mobilisation to improve health outcomes and reduce rates of VTE.

## Introduction

Primary total hip arthroplasty (THA) and total knee arthroplasty (TKA) are commonly performed procedures with over 100,000 hip and knee replacements performed in Australia in 2015 [1]. They are recognised as effective interventions to restore limb function and alleviate pain associated with hip or knee arthritis [2]. However, the risk of venous thromboembolism (VTE) developing post-operatively remains a significant health concern. A proposed intervention to reduce rates of VTE is early mobilisation of patients following surgery; it is recommended in VTE prevention guidelines [3–6] and advocated in fast-track surgical protocols for THA and TKA [7, 8]. In the latter, mobilisation commences the same day of surgery.

Early mobilisation, or early ambulation, provides a range of health benefits beyond its potential prevention of VTE. A systematic review of early mobilisation protocols in various medical and surgical subspecialties found reduced rates of VTE and other post-operative complications such as pneumonia, atelectasis, urinary tract infections, sepsis, myocardial infarction and stroke [9]. Fast-track pathways for TKA and THA, where early mobilisation is a key feature, have reported reduced length of stay (LOS), reduced post-operative morbidity and mortality, improved patient satisfaction, and reduced VTE and its sequelae [8, 10–12].

Despite widespread endorsement of early mobilisation in the arthroplasty literature, there is currently no accepted definition. Aspects such as the ideal time to initiate mobilisation (e.g. post-operative day 0 [POD 0], POD 1) remains unclear and often left to the discretion of hospital staff [13]. This leads to practice variation and a potential lack of best practice. Notably, fast-track protocols initiate mobilisation within 2–4 hours post-operatively without apparent detriment to the patient, suggesting that mobilisation on POD 0 is safe and feasible [8, 12, 14].

The overarching aim of this study was to identify how early mobilisation is interpreted by Australian arthroplasty service providers following THA and TKA. The objectives were to determine: (1) the proportion of patients that first mobilised on POD 0, and (2) factors associated with an earlier time to mobilisation.

## Materials and methods

### Setting and study design

This was a sub-study conducted within a larger prospective observational study, EPOC (Evidence-based Processes and Outcomes of Care), an ongoing multicentre study concerned with acute-care and long-term outcomes for patients undergoing primary THA or TKA (ClinicalTrials.gov NCT01899443). The EPOC study had a part-random, part-convenience selection of 19 Australian sites identified through the National Joint Replacement Registry. The selection criterion was that each site must be a high-volume arthroplasty provider, defined as exceeding 275 hip or knee arthroplasties in 2012. Consecutive THA and TKA patients were enrolled between August 2013 and January 2015. Ethical and governance approvals were obtained from several Human Research and Ethics Committees (HRECs): Hunter New England HREC (NSW); St Vincent’s Health and Aged Care HREC (Queensland); The Prince Charles Hospital HREC (Queensland); Austin Health HREC (Victoria); Barwon Health HREC (Victoria); Epworth HREC (Victoria); Calvary Health Care Clinical and Research Ethics Committee (Tasmania and Riverina); and Calvary Healthcare Adelaide HREC (South Australia). Written informed consent was obtained from all eligible patients who agreed to participate.

The inclusion criteria for this sub-study were patients aged 18 years and older, osteoarthritis as the primary diagnosis and indicator for surgery, primary elective unilateral THA or TKA, and capable of providing informed consent. Those with cognitive impairment, undergoing revision THA or TKA, undergoing arthroplasty for other diagnoses (e.g. fracture, avascular necrosis) or presenting for arthroplasty of a second joint, but already recruited into the study, were excluded.

### Data sources and variables

Hospital sites prospectively collected baseline, peri-operative and treatment-related patient data. For quality control purposes, the data submitted were audited by researchers via review of all medical records (paper-based and electronic) pertaining to the index episode of care. Patient-related variables included age, gender, body mass index (BMI), education level, comorbidities, American Society of Anaesthesiologists (ASA) score and pre-operative patient-reported outcome measures (PROMs). ASA score is a quantifiable measure of health status ranging from 1 (normal healthy patient) to 5 (moribund patient not expected to survive). Pre-operative PROMs included the EuroQol-5D visual analogue scale (EQ-5D VAS), which measures the patient’s self-rated health on a scale from 0 (“worst imaginable health state”) to 100 (“best imaginable health state”) [15], and Oxford Hip Score (OHS) or Oxford Knee Scores (OKS), which captures joint-specific pain and function on a scale from 0 (worst outcome/most symptoms) to 48 (best outcome/least symptoms) [16].

Treatment-related variables included hospital site, hospital status (public hospital/private hospital), surgical hip approach (for THA), anaesthetic details, local infiltration analgesia (LIA), intra-articular drainage, indwelling catheter (IDC), donor blood transfusion, the presence of an acute complication and LOS. Acute surgical complications were categorised using the original and a modified version of the Clavien-Dindo (CD) classification, which is based on the type of therapy necessary to treat the complication [17]. Two authors independently categorised each complication into the original or modified CD classification. Any disagreements were resolved through discussion and consensus, and the consultation of a third author. Our modified CD was designed to capture complications known clinically to affect early mobilisation, such as excessive pain and swelling, headaches or migraines, nausea or vomiting [2], symptomatic anaemia or symptomatic hypotension [14], hypertension or labile blood pressure, and gastrointestinal symptoms (see S1 Table for full list of complications), which are excluded from the original CD classification. Our modified CD did not include asymptomatic anaemia as a complication, as it can be deemed a reasonable sequela after major surgery. Additionally, the presence of donor blood transfusion (which can be considered due to severe symptomatic anaemia) was regarded as a separate potential covariate influencing early mobilisation. LOS was determined as the length of time from the date of surgery until the date of discharge from the orthopaedic ward.

### Outcome measures

The primary outcome of interest was the first post-operative day of mobilisation (i.e. the first post-operative day that patients walked, including any partial or full weight-bearing activities such as walking on the spot, bed-to-chair and bed-to-toilet). Patients that began mobilising on the day of surgery, before midnight, were recorded under POD 0. Patients that mobilised the following day were recorded under POD 1, and so forth. The data were recorded on study-specific standardised forms by hospital site staff. We checked the accuracy and completeness of what was submitted on the standardised forms against the medical records (including paper and electronic entries).

### Statistical analysis

Simple descriptive statistics, including means, medians, quartiles and proportions, were used to describe the overall and joint-specific cohorts and highlight population characteristics and the proportion of patients first mobilising on POD 0.

Statistical methods that allowed simultaneous consideration of multiple factors with an outcome of interest were used to identify associations between covariates and mobilisation outcomes. Poisson regression was used, as the primary outcome was count data. All patient- and treatment-related variables were included in regression analysis: age (separated into quartiles), gender, education level (none/year 10 and below/year11-12, trade certificate, diploma/graduate degree), comorbidities (none/≥1 without daily medication/≥1 with daily medication), BMI (separated into categories typically used to describe levels of obesity [<25kg/m^2^, 25–29.9kg/m^2^, 30–34.9kg/m^2^ and ≥35kg/m^2^]), ASA score, pre-operative EQ-5D VAS (separated into above or below the median of 75), pre-operative OHS/OKS (separated into categories based on severity of joint impairment or symptoms [0–12, extreme to severe/13-24, severe to moderate/25-36, moderate to mild/37-48, mild to nil impairment or symptoms]), hospital site (several of the smaller 19 hospital sites were combined into one for analysis, resulting in 15 sites in total), hospital status (public hospital/private hospital), spinal or epidural block, regional nerve block, anterior hip approach (yes/other hip approach), LIA, intra-articular drain, IDC, presence of an acute complication and donor blood transfusion. Age was categorised into quartiles to provide more clinically meaningful information, as statistical analysis calculated the risk ratio (RR) to determine the quantitative effects of each covariate. As age is normally a continuous variable, the RR would refer to an increment of one single year, which is less meaningful in a clinical setting. Similar reasons applied to categorising other numerical variables such as BMI and OHS/OKS for statistical analysis. We identified missing data as missing completely at random, and performed a complete case analysis approach (excluding patients with missing information), as we believed the missing data would have minimal influence on the analysis results.

Independent variables individually associated with the outcome with a value of P ≤ 0.20 on univariate Poisson analyses were selected for a backward stepwise Poisson regression model respectively. Backward stepwise regression was chosen given the exploratory nature of the analyses. A two-tailed value of P < 0.05 was considered significant and retained in the final backward stepwise regression models. RR was calculated in Poisson regression with 95% confidence intervals (CI) to determine the quantitative effects of each covariate. Log Poisson regression was used to calculate RR. THA and TKA patients were analysed separately. All statistical analyses were performed using SAS (version 9.4). This observational study was reported according to the STROBE guidelines.

The sample size for this nested study was dictated by the sample size (approximately 2000) for the main study, EPOC, which aimed to determine the relationship between receiving care that complies with guidelines and risk of a composite adverse event outcome. However, a post-hoc power calculation was performed to determine the power of this nested study. A minimum of 750 patients undergoing THA would provide 80% power (at 5% significance level) to detect a possible RR of 1.55 for a variable of interest associated with early mobilisation, assuming that the early mobilisation rate (mobilising POD 0 or POD 1) is about 60%. The same calculation applies for a minimum of 750 patients undergoing TKA.

## Results

### Population characteristics

**Fig 1:**
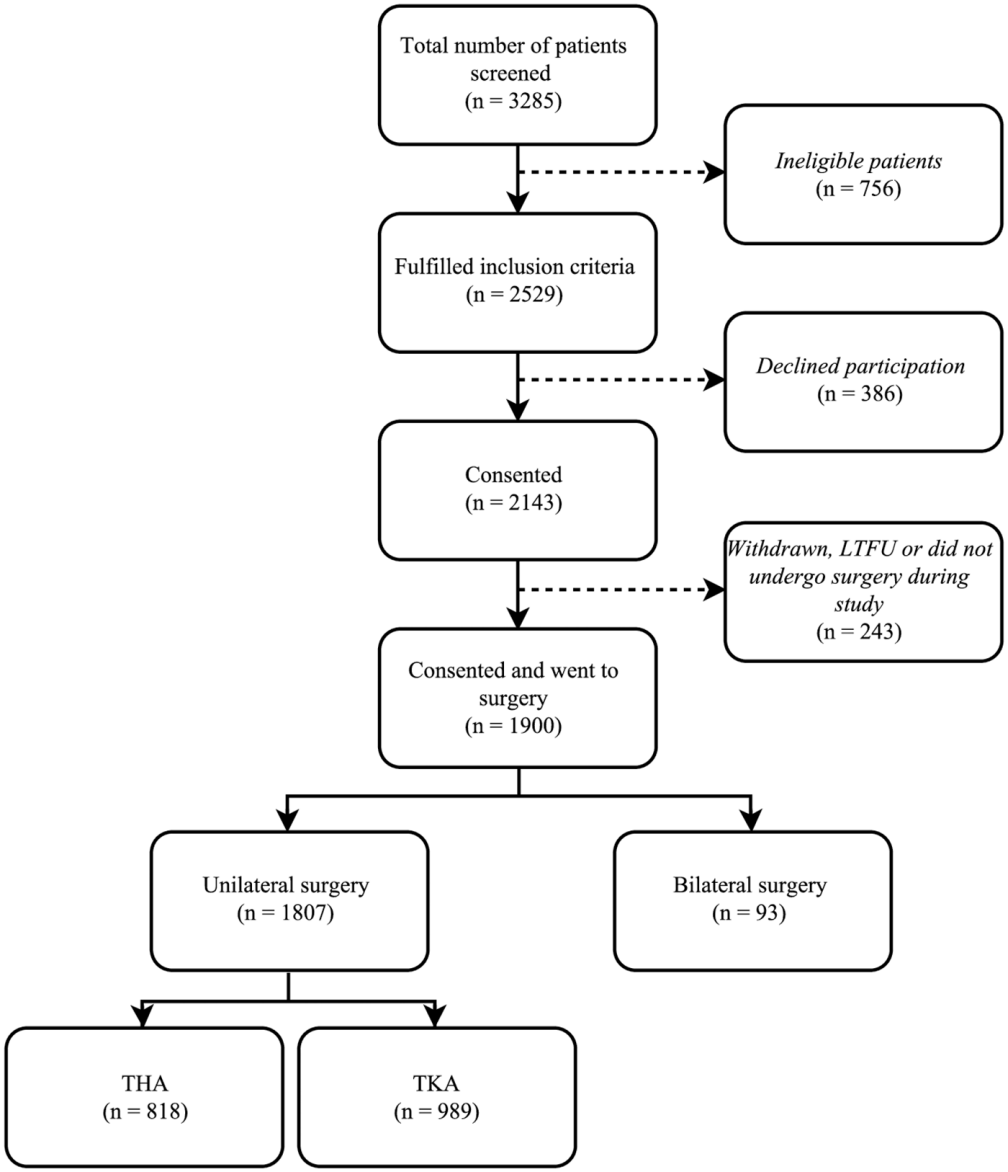
Flow diagram for study participation. LTFU = loss to follow up; THA = total hip arthroplasty; TKA = total knee arthroplasty.

In the EPOC study, 3285 patients were screened, 2529 met the inclusion criteria, and 1900 consented and underwent surgery. Of these, 1807 patients underwent unilateral surgery and 93 underwent bilateral surgery. The 1807 unilateral patients were included in this study (Fig 1). The distribution of patient- and treatment-related characteristics for THA (n = 818) and TKA (n = 989) patients are shown in Table 1. In general, patients were elderly (mean age ± SD, 68.3 ± 8.7 years THA, 66.9 ± 9.7 years TKA), female (52.7% THA, 56.3% TKA) and overweight (mean BMI ± SD, 29.2 ± 5.8 kg/m^2^ THA, 32.1 ± 6.6 kg/m^2^ TKA). The most common comorbidities were gastrointestinal reflux, hypercholesterolaemia and hypertension.

**Table 1:** Distribution of patient- and treatment-related characteristics of THA and TKA patients. Values are presented as N (%) unless indicated otherwise.

Parameter	THA (n = 818)	TKA (n = 989)
**Patient-related characteristics**		
Age, years		
• Quartile 1 (THA 18–58, TKA 18–62)	200 (24.5)	236 (23.9)
• Quartile 2 (THA 59–65, TKA 63–67)	191(23.3)	216 (21.8)
• Quartile 3 (THA 66–72, TKA 68–74)	217 (26.5)	286 (28.9)
• Quartile 4 (THA ≥73, TKA ≥75)	210 (25.7)	251 (25.4)
Female sex	431 (52.7)	557 (56.3)
BMI, kg/m^2^		
• < 25	205 (25.1)	110 (11.1)
• 25–29.9	304 (37.3)	321 (32.6)
• 30–34.9	183 (22.5)	291 (29.5)
• ≥ 35	123 (15.1)	264 (26.8)
Education level		
• No education	0 (0)	5 (0.5)
• Year 10 and below	228 (28.3)	403 (41.2)
• Year 11-12/trade certificate/diploma	428 (53.1)	471 (48.2)
• Graduate degree	150 (18.6)	99 (10.1)
Comorbidities		
• None	131 (16.0)	81 (8.1)
• ≥ 1 without daily medication	81 (9.9)	46 (4.6)
• ≥ 1 with daily medication	606 (74.1)	862 (87.2)
ASA Score		
• ASA I	107 (13.3)	73 (7.6)
• ASA II	487 (60.3)	519 (54.2)
• ASA III	204 (25.3)	349 (36.4)
• ASA IV	9 (1.1)	16 (1.7)
• ASA V	0 (0)	1 (0.1)
Pre-operative EQ-5D VAS, no. above median (%)	445 (54.9)	517 (52.9)
Pre-operative OHS/OKS		
• 0–12	156 (19.3)	136 (13.9)
• 13–24	368 (45.7)	490 (50.3)
• 25–36	238 (29.5)	301 (30.9)
• 37–48	44 (5.5)	48 (4.9)
**Treatment-related characteristics**		
Hospital site		
• Site 1	92 (11.2)	200 (20.2)
• Site 2	19 (2.3)	32 (3.2)
• Site 3	118 (14.4)	58 (5.9)
• Site 4	18 (2.2)	51 (5.2)
• Site 5	23 (2.8)	56 (5.7)
• Site 6	93 (11.4)	17 (1.7)
• Site 7	31 (3.8)	24 (2.4)
• Site 8	61 (7.5)	61 (6.2)
• Site 9	24 (2.9)	20 (2.0)
• Site 10	17 (2.1)	46 (4.6)
• Site 11	101 (12.3)	145 (14.7)
• Site 12	58 (7.1)	39 (3.9)
• Site 13	76 (9.3)	120 (12.1)
• Site 14	33 (4.1)	66 (6.7)
• Site 15	54 (6.6)	54 (5.5)
Public hospital status	314 (38.4)	538 (54.4)
Anaesthetic details		
• General anaesthetic	539 (66.0)	572 (57.8)
• Sedation	265 (32.4)	376 (38.0)
• Spinal or epidural block	458 (56.1)	649 (65.6)
• Regional nerve block	80 (9.8)	248 (25.1)
Hip surgical approach		
• Anterior hip approach^1^	254(31.1)	-
• Anterolateral/modified Hardinge	26 (3.2)	-
• Lateral/Hardinge	26 (3.2)	-
• Posterior/posterolateral	501(61.5)	-
• Superpath	8 (1.0)	-
Tourniquet	-	851 (86.7)
Local infiltration analgesia	639 (78.7)	715 (72.8)
Intra-articular drainage	299 (36.6)	479 (48.5)
Indwelling catheter	593 (72.6)	798 (80.7)
Donor blood transfusion	52 (6.5)	48 (4.9)
Acute complication original CD	146 (18.0)	288 (29.3)
Acute complication modified CD	267 (32.9)	387 (39.3)
Length of stay, mean ± SD	5.0 ± 2.4	6.1 ± 3.1

THA = total hip arthroplasty; TKA = total knee arthroplasty; BMI = body mass index; ASA = American Society of Anaesthesiologists; EQ-5D VAS = EuroQol-5D visual analogue scale; OHS = Oxford hip score; OKS = Oxford knee score; CD = Clavien-Dindo; SD = standard deviation.

^1^Anterior hip approaches include direct anterior approach (DAA), anterior minimally invasive surgery (AMIS) and Smith-Peterson

The majority of operations (58% of the entire cohort) started in the morning, with 41% completed before 12 noon. Most patients had an IDC and no intra-articular drainage. For THA procedures, the posterior hip approach was most common (61.5%), followed by the anterior hip approach (31.1%). Using the original CD classification, 18.0% of THA and 29.3% of TKA patients experienced an acute complication. Using our comparatively sensitive modified CD classification, acute complications increased to 32.9% and 39.3% respectively. Three patients died post-operatively due to acute complications. Our modified CD showed a total of 936 complications occurred in 698 patients. The most common complications were minor or transient and included symptomatic anaemia or hypotension (27.8% of patients with a complication) followed by additional antibiotic treatment for any infective condition (19.9%) (see S2 Table for full list of frequency of complications).

### First day of mobilisation

**Fig 2:**
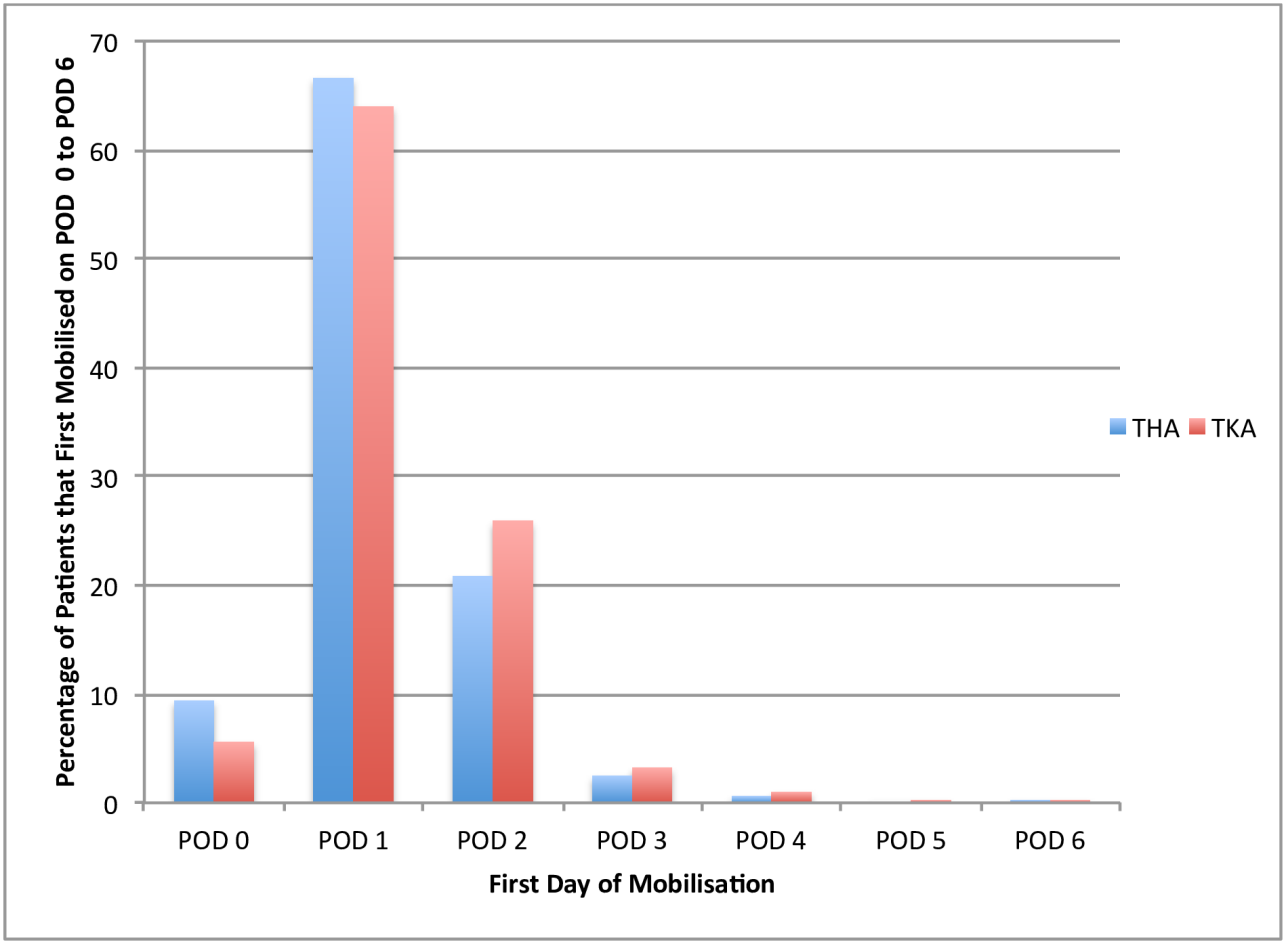
The percentage of patients that first mobilised on POD 0 to POD 6.

Only 9.4% of THA and 5.6% of TKA patients first mobilised on POD 0 (Table 2) (Fig 2). By the end of POD 1, 76.0% of THA and 69.6% of TKA had mobilised. By the end of POD 6, the entire cohort had mobilised.

**Table 2:** First day of mobilisation for THA and TKA patients. Values are presented as N (%) unless indicated otherwise.

	Post-operative day						
	POD 0	POD 1	POD 2	POD 3	POD 4	POD 5	POD 6
**THA**	76 (9.4)	538 (66.6)	168 (20.8)	20 (2.5)	5 (0.6)	-	1 (0.1)
**TKA**	55 (5.6)	629 (64.0)	254 (25.9)	31 (3.2)	10 (1.0)	2 (0.2)	1 (0.1)
**Total**	131 (7.3)	1167 (65.2)	422 (23.6)	51 (2.9)	15 (0.8)	2 (0.1)	2 (0.1)

POD = post-operative day.

### Factors associated with earlier time to mobilisation

For THA patients, univariate analysis found many variables associated with an earlier time to mobilisation including several hospital sites (3 of 15 sites), anterior hip approach, no spinal or epidural block, no intra-articular drainage, no IDC, no acute complication and no donor transfusion (Table 3). However, the majority of these variables associated with earlier time to mobilisation were not significant after multivariate analysis. Earlier time to mobilisation was more likely to occur in patients without an IDC (RR 1.38, 95% CI 1.09–1.73, p = 0.006), without an acute complication (RR 1.20, 95% CI 1.04–1.38, p = 0.013) and more likely to occur in several hospital sites (4 of 15 sites) (Table 3).

**Table 3:** Poisson regression analyses for factors associated with time to mobilisation*.

	THA		TKA	
Parameter	Univariate RR (95% CI)	Multivariate RR (95% CI)	Univariate RR (95% CI)	Multivariate RR (95% CI)
**Patient-related characteristics**				
Age				
• Quartile 1	1.00	-	1.00	-
• Quartile 2	0.98 (0.82–1.18)	-	1.02 (0.87–1.20)	-
• Quartile 3	1.04 (0.87–1.25)	-	1.00(0.86–1.17)	-
• Quartile 4	1.05 (0.88–1.25)	-	1.05 (0.90–1.22)	-
Gender				
• Female	1.00	-	1.00	-
• Male	0.90 (0.79–1.02)	-	0.88 (0.79–0.99)†	-
BMI				
• < 25	1.00	-	1.00	-
• 25–29.9	0.94 (0.80–1.11)	-	1.09 (0.89–1.33)	-
• 30–34.9	0.99 (0.82–1.19)	-	1.14 (0.93–1.39)	-
• ≥ 35	1.01 (0.82–1.24)	-	1.21 (0.99–1.48)	-
Education level				
• No education	-	-	0.93 (0.42–2.08)	-
• Year 10 and below	1.00	-	1.00	-
• Year 11-12/trade certificate/diploma	0.97 (0.83–1.12)	-	1.03(0.92–1.16)	-
• Graduate degree	0.94 (0.77–1.13)	-	0.98 (0.80–1.19)	-
Comorbidities				
• None	1.00	-	1.00	-
• ≥ 1 without daily medication	1.02 (0.79–1.33)	-	1.02(0.73–1.41)	-
• ≥ 1 with daily medication	1.07 (0.90–1.28)	-	1.11 (0.90–1.36)	-
ASA Score				
• ASA I	1.00	-	1.00	-
• ASA II	1.01 (0.83–1.24)	-	1.08 (0.86–1.34)	-
• ASA III	1.11 (0.90–1.38)	-	1.12 (0.89–1.41)	-
• ASA IV	1.17 (0.64–2.11)	-	1.30 (0.83–2.02)	-
• ASA V	-	-	1.66 (0.41–6.74)	-
Pre-operative EQ-5D VAS				
• Below median (< 75)	1.00	-	1.00	-
• Median and above (≥ 75)	0.98 (0.86–1.12)	-	0.95 (0.85–1.06)	-
Pre-operative OHS/OKS				
• 0–12	1.00	-	1.00	-
• 13–24	0.89 (0.75–1.05)	-	1.04 (0.88–1.22)	-
• 25–36	0.90 (0.75–1.08)	-	1.01 (0.84–1.21)	-
• 37–48	0.95 (0.70–1.28)	-	0.91 (0.67–1.22)	-
**Treatment-related characteristics**				
Hospital site				
• Site 1	1.00	1.00	1.00	1.00
• Site 2	0.94 (0.61–1.44)	0.90 (0.58–1.38)	0.79 (0.56–1.12)	0.79 (0.56–1.12)
• Site 3	0.50 (0.38–0.66)§	0.68 (0.48–0.95)†	0.78 (0.60–1.02)	0.78 (0.60–1.02)
• Site 4	0.28 (0.13–0.59)§	0.33 (0.15–0.71)‡	0.46 (0.32–0.65)§	0.46 (0.32–0.54)§
• Site 5	0.77 (0.50–1.19)	0.72 (0.47–1.11)	0.76 (0.58–0.99)†	0.74 (0.56–0.97)†
• Site 6	0.83 (0.64–1.07)	0.83 (0.64–1.08)	0.77 (0.48–1.22)	0.74 (0.45–1.20)
• Site 7	1.09 (0.77–1.54)	1.24 (0.85–1.81)	0.95 (0.66–1.37)	0.96 (0.66–1.39)
• Site 8	1.06 (0.81–1.39)	1.05 (0.80–1.38)	1.31 (1.06–1.63)†	1.33 (1.07–1.66)†
• Site 9	0.74 (0.48–1.14)	0.68 (0.44–1.05)	0.72 (0.46–1.12)	0.73(0.47–1.13)
• Site 10	0.59 (0.34–1.02)	0.67 (0.38–1.18)	0.48 (0.33–0.69)§	0.48 (0.33–0.69)§
• Site 11	1.20 (0.95–1.51)	1.16 (0.92–1.47)	1.24 (1.05–1.46)†	1.23(1.04–1.46)†
• Site 12	0.77 (0.57–1.04)	0.72 (0.53–0.99)†	0.77 (0.56–1.06)	0.77(0.55–1.09)
• Site 13	0.81 (0.62–1.07)	0.87 (0.65–0.16)	0.83 (0.68–1.02)	0.82 (0.67–0.99)†
• Site 14	0.65 (0.43–0.96)†	0.60 (0.40–0.89)†	0.70 (0.53–0.91)‡	0.69 (0.53–0.90)‡
• Site 15	0.88 (0.65–1.19)	0.90 (0.65–1.23)	0.81 (0.61–1.06)	0.78 (0.59–1.04)
Hospital status				
• Public hospital	1.00	-	1.00	-
• Private hospital	0.97 (0.85–1.10)	-	1.14 (1.02–1.27)†	-
Hip approach				
• Other hip approaches^1^	1.00	-	-	-
• Anterior hip approach^2^	0.82 (0.72–0.95)‡	-	-	-
Spinal or epidural block				
• No	1.00	-	1.00	-
• Yes	1.21 (1.06–1.38)‡	-	1.04 (0.93–1.17)	-
Regional nerve block				
• No	1.00	-	1.00	-
• Yes	1.00 (0.81–1.24)	-	0.98 (0.87–1.12)	-
Local infiltrative analgesia				
• No	1.00	-	1.00	-
• Yes	0.92 (0.79–1.07)	-	0.89 (0.79–1.01)	-
Intra-articular drainage				
• No	1.00	-	1.00	-
• Yes	1.16 (1.02–1.32)†	-	1.10 (0.98–1.22)	-
Indwelling catheter				
• No	1.00	1.00	1.00	-
• Yes	1.59 (1.35–1.87)§	1.38 (1.09–1.73)‡	1.34 (1.17–1.58)§	-
Donor blood transfusion				
• No	1.00	-	1.00	1.00
• Yes	1.27 (1.00–1.61)†	-	1.30 (1.03–1.62)†	1.29 (1.03–1.63)†
Acute complication (modified CD)				
• No	1.00	1.00	1.00	-
• Yes	1.20 (1.05–1.37)‡	1.20 (1.04–1.38)†	1.07 (0.96–1.20)	-

THA = total hip arthroplasty; TKA = total knee arthroplasty; RR = risk ratio; 95% CI = 95% confidence interval; BMI = body mass index; ASA = American Society of Anaesthesiologists; EQ-5D VAS = EuroQol-5D visual analogue scale; OHS = Oxford hip score; OKS = Oxford knee score; DAA = direct anterior approach; AMIS = anterior minimally invasive surgery; CD = Clavien-Dindo.

^†^ P < 0.05

^‡^ P < 0.01

^§^ P < 0.001

^1^Other hip approaches include anterolateral/modified Hardinge, lateral/Hardinge, posterior/posterolateral and Superpath

^2^Anterior hip approaches include direct anterior approach (DAA), anterior minimally invasive surgery (AMIS) and Smith-Peterson

For TKA patients, univariate analysis found many variables associated with earlier time to mobilisation including male gender, several hospital sites (6 of 15 sites), public hospitals, no IDC and no donor transfusion (Table 3). However, only two of these variables remained significant after multivariate analysis. Earlier time to mobilisation was more likely in patients without a donor blood transfusion (RR 1.29, 95% CI 1.03–1.63, p = 0.028) and more likely in several hospital sites (7 of 15 sites) (Table 3).

## Discussion

Early mobilisation is frequently advocated after THA or TKA, yet there is inadequate information regarding the ideal time to begin mobilisation and which factors could be manipulated in order to encourage or permit earlier mobilisation. This novel study provides a snapshot of current practice in Australia on how early mobilisation after THA or TKA is interpreted and explores what factors may influence the post-operative day of mobilisation.

We observed that very few patients (<10%) mobilised on POD 0, despite a large proportion of operations (41%) completed before 12 noon, which theoretically should have provided adequate time to initiate mobilisation as per fast-track protocols [8, 12, 18, 19]. To our knowledge, mobilisation on POD 0 is safe and feasible. Studies with fast-track protocols for THA and TKA have initiated mobilisation for all patients on POD 0 [8, 12, 18, 19], or compared mobilisation on POD 0 with POD 1 [13, 20–22], with none reporting adverse events related to the earlier time.

In terms of time to mobilisation after THA or TKA, ‘hospital site’ was a significant covariate. This perhaps reflects hospital site protocols, as certain hospitals deliberately mobilised patients on POD 0 whilst other hospitals did not. Differences in mobilisation protocols may reflect uncertainty in the literature concerning what is meant by early mobilisation, but site resource constraints might also contribute to between-hospital protocol variations, as early mobilisation after surgery is resource intensive and often dependent on staff availability, which can be lacking on weekends or late in the evening [14, 21, 23]. The presence of an IDC was associated with delayed mobilisation after THA, which may reflect the mechanical interference of IDCs with walking and the existing hospital culture that begins mobilising patients to the bathroom after IDC removal for voiding of bladders. The presence of an acute complication was associated with delayed mobilisation after THA, which supports previous claims that post-operative complications such as pain, nausea, vomiting and hypotension can limit and delay mobilisation after THA or TKA [2, 14]. However, our results contrast another study, which found pain or nausea and vomiting were not associated with the patient’s ability to undergo physiotherapy on POD 0 after THA or TKA [21]. Our finding that donor blood transfusion was associated with delayed mobilisation after TKA supports previous research that blood transfusion was associated with a lack of mobilisation on POD 0 after THA or TKA [24]. Likely reasons are that blood transfusions can take several hours, which delays mobilisation, and that patients requiring transfusion are unlikely to mobilise due to severe symptomatic anaemia. LIA should theoretically assist with full-weight bearing post-operatively, as a systematic review and meta-analyses reported that LIA improves early analgesia after THA or TKA in the 48-hour post-operative period [25, 26]. However, we found no significant association between LIA and our outcomes on multivariate analysis.

Interestingly, our multivariate analysis found no association between time to mobilisation and anaesthetic blocks, or THA patients receiving an anterior hip approach. Regional anaesthesia is known to cause a motor deficit [27], which could reasonably limit one’s ability to ambulate. However, our findings suggest that there is no relation between this factor and how early patients begin to mobilise. Additionally, the anterior hip approach has faster recovery in the first 2 weeks after surgery [28], which has been attributed to minimal muscle damage and soft tissue trauma with the anterior approach when compared with standard anterolateral or posterolateral approaches [29, 30]. Our findings suggest that there is no association between this hip approach and the time period for patients to first begin mobilising. We believe these findings reflect the major influence of hospital site, with hospital staff mobilising patients based on local protocol, irrespective of the type of surgical hip approach that was performed.

Our study found no patient-related factors were associated with time to mobilisation on multivariate analysis. This finding is indirectly supported by an earlier study that concluded on univariate analysis that age, BMI, number of comorbidities and ASA score were not associated with timing and the ability to undergo physiotherapy on POD 0 after THA or TKA [21].

Our study has several strengths. We described mobilisation outcomes for two large prospective cohorts of primary unilateral THA and TKA recipients with end-stage osteoarthritis. Our audit of all acute medical records for quality control provided the opportunity for clarification, accuracy checking and completion of missing data where required. Over 1000 data points per patient (though not all used) were collected to comprehensively account for many potential confounders. Furthermore, we focused on high-volume sites to reduce the ‘noise’ created by including sites relatively inexperienced in arthroplasty surgery, and we included both private and public sector providers from five Australian states to increase our generalisability to highly experienced centres nationally.

Several limitations were identified with our study. Firstly, no causal conclusions can be inferred from the observational study design. Secondly, we modified the Clavien-Dindo classification to include excessive pain and swelling, headaches or migraines, nausea or vomiting, symptomatic anaemia or symptomatic hypotension, hypertension or labile blood pressure, and gastrointestinal symptoms. We recognise that the properties of our modified CD classification are not previously established in literature, however, we believe these modifications are justified, given that these additional post-operative conditions are known clinically to affect early mobilisation. Thirdly, another potential limitation is that we recorded time to mobilisation in post-operative days and not hours after surgery. This may misrepresent how early patients mobilised, as those with afternoon operations who mobilised the following morning would have mobilised within a 24-hour window, suggesting a more aggressive mobilisation protocol than our data implies. However, if sites were mobilising patients based on hours post-surgery and not on a day-by-day protocol, we would have observed patients with morning operations being mobilised on the afternoon of surgery. This was not observed despite a large proportion of operations (41%) completed before 12 noon, thus we contend that Australian arthroplasty sites most typically commence mobilisation on POD 1. Finally, we only reported when patients commenced mobilisation, and not how much mobilisation is typically performed. We had originally intended to record the distances that patients mobilised on each post-operative day. However, we removed this secondary outcome from the study, as it was inadequately recorded on our data collection form and appeared prone to bias.

Future research might explore issues we identified, including whether THA and TKA patients are mobilised at appropriate times and whether early mobilisation is an effective intervention for improving health outcomes and reducing rates of VTE. Further aspects include whether early mobilisation activities are adequate, such as walking a clinically appropriate distance or performing specific rehabilitation exercises. Despite recommendation of early mobilisation for VTE prevention, no randomised controlled trials have investigated this relationship. Nevertheless, the EPOC study was designed in part to explore this relationship in the context of other variables (e.g. anticoagulants, stockings) and will report these findings in due course.

## Conclusion

In contrast to recommendations in fast-track protocols, few THA and TKA patients (<10%) mobilise on POD 0 (the day of surgery) in Australian hospitals. For THA patients, earlier time to mobilisation was associated with hospital site, and absences of an indwelling catheter and acute complications. For TKA patients, earlier time to mobilisation was associated with hospital site and absence of donor blood transfusion. Encouragingly, treatment-related factors are associated with POD of mobilisation, indicating the potential pivotal role of service providers in promoting early mobilisation, especially if earlier mobilisation is shown to improve health outcomes and reduce adverse events.

## Supporting information

S1 Table This is the modified Clavien-Dindo classification used. (DOCX)

S2 Table This is the frequency of Acute Surgical complications. (DOCX)
