# Supplementary material for: Early mobilisation after total hip or knee arthroplasty: A multicentre prospective observational study
Source: PLoS One. 2017 Jun 27;12(6):e0179820. doi: 10.1371/journal.pone.0179820 (PMC5487040; doi:10.1371/journal.pone.0179820)
Supplement: S1 Table — (DOCX) [file pone.0179820.s001.docx]

**S1 Table. The Modified Clavien-Dindo Classification Used**

| **Grade** | **Modified Clavien-Dindo Classification of Surgical Complications** |
| --- | --- |
| I | **Any deviation from the normal post-operative course without the need for pharmacological treatment or operative treatment (surgical, endoscopic, radiological).**   - **Includes antiemetics, antipyretics, analgesics, diuretics, electrolytes and physiotherapy.** |
| Examples | **The original Clavien-Dindo classification**  Abnormal liver function tests (withheld clexane), adverse drug reaction/drug allergy, atelectasis, bladder retention, blister, delirium/post-op confusion, electrolyte imbalance, excessive bleeding, gluteal tendinopathy, haematuria, haematoma, fall, neuropraxia, phlebitis, physiotherapy (Zimmer splint), pressure sore, superficial corneal abrasion (ointment)  **The additional complications included in our modified Clavien-Dindo classification**  Excessive pain or swelling; headaches or migraines; nausea or vomiting; symptomatic anaemia or symptomatic hypotension; hypertension or labile blood pressure; gastrointestinal symptoms |
| **II** | **Requiring pharmacological treatment (drugs other than allowed in Grade I)**   - **Includes antibiotics, transfusions or total parenteral nutrition** - **Did NOT include blood transfusions** |
| Examples | Additional antibiotic treatment (urinary tract infection, respiratory infection, gastrointestinal infection, skin [oozy wound, blister, superficial surgical site infection, cellulitis], deep surgical site infection, joint infection, other), arrhythmia (atrial fibrillation [AF], ventricular fibrillation, ‘heart flutter’), dehiscence (no surgery), DVT, gout flare, hyperglycaemia, infusion (iron, albumex), pulmonary oedema, transient ischaemic attack |
| **III** | **Requiring operative treatment (surgical, endoscopic, radiological)**   - **IIIa: operation not under general anaesthesia** - **IIIb: operation under general anaesthesia** |
| Examples | Dehiscence, dislocation, intraoperative complications (fracture, medial collateral ligament [MCL] strain or avulsion, patellar tendon nick or rupture), pleural effusion, reoperation (epidural blood patch) |
| **IV** | **Life-threatening complication (including CNS, excludes TIA) requiring ICU/HDU (unplanned)**   - **IVa: single organ dysfunction** - **IVb: multi organ dysfunction** |
| Examples | Cardiac complications (acute myocardial infarction [AMI, unplanned ICU admission due to arrhythmia, AF, raised troponins], acute congestive cardiac failure [CCF]), central nervous system [CNS] complications (seizure, stroke), respiratory complications (PE, respiratory depression), renal complications (acute kidney injury), unplanned ICU admissions (haemodynamic instability, hypotension, drug toxicity) |
| **V** | **Mortality (death)** |
