# Supplementary material for: Early mobilisation after total hip or knee arthroplasty: A multicentre prospective observational study
Source: PLoS One. 2017 Jun 27;12(6):e0179820. doi: 10.1371/journal.pone.0179820 (PMC5487040; doi:10.1371/journal.pone.0179820)
Supplement: S2 Table — (DOCX) [file pone.0179820.s002.docx]

**S2 Table. Frequency of Acute Surgical Complications**

The high-sensitivity modified Clavien-Dindo (CD) classification showed a total of 936 complications occurred in 698 THA and TKA patients. In these 698 patients, the frequency of specific complications is detailed below, *e.g. 31.38% of all patients with an acute complication (n = 698) had a CD Grade I complication*. The cumulative total is more than 100%, as some patients had more than one complication.

| **Grade** | **Frequency of Acute Surgical Complications** | |
| --- | --- | --- |
| I | All Original CD I Complications (31.38%)  8.17% bladder retention  5.30% delirium  3.15% fall  3.01% blister  2.72% pressure sore  1.86% adverse drug reaction  1.43% atelectasis  1.15% electrolyte imbalance  1.00% neuropraxia  0.86% haematuria  0.86% haematoma  0.72% physiotherapy intervention  0.43% abnormal liver function tests  0.29% excessive bleeding  0.14% gluteal tendinopathy  0.14% phlebitis  0.14% superficial corneal abrasion | All Additional Modified CD I Complications (60.6%)  27.79% symptomatic anaemia or symptomatic hypotension  13.32% excessive pain or swelling  12.32% nausea or vomiting (without antiemetics)  3.15% gastrointestinal symptoms  2.87% hypertension or labile blood pressure  1.15% headache or migraines |
| II | All Original CD II Complications (31.23%)  19.91% additional antibiotic treatment for any infective condition  4.87% arrhythmia (AF, VF, ‘heart flutter’)  4.15% DVT or thrombosis  0.57% pulmonary oedema  0.57% infusion (iron, albumex)  0.43% transient ischaemic attack  0.29% gout flare  0.29% hyperglycaemia  0.14% dehiscence (without surgery) | |
| III | All Original CD III Complications (3.72%)  1.58% intraoperative fracture  0.86% other intraoperative complications (MCL strain or avulsion, patellar tendon nick or rupture)  0.72% dehiscence  0.29% pleural effusion  0.14% dislocation  0.14% reoperation | |
| IV | All Original CD IV Complications (7.16%)  2.15% heart complications (AMI, CCF, unplanned ICU admission due to arrhythmia, AF, raised troponins)  1.72% unplanned ICU admission (haemodynamic instability, hypotension, drug toxicity)  1.58% respiratory complications (PE, respiratory depression)  1.29% kidney complications (AKI)  0.43% CNS complications (seizure, stroke) | |
| V | All Original CD V Complications (0.43%)  0.43% death | |
